# Triggering of Suicidal Erythrocyte Death by Celecoxib

**DOI:** 10.3390/toxins5091543

**Published:** 2013-09-10

**Authors:** Adrian Lupescu, Rosi Bissinger, Kashif Jilani, Florian Lang

**Affiliations:** Department of Physiology, University of Tuebingen, Gmelinstraße 5, Tuebingen 72076, Germany; E-Mails: lupescuadrian@gmx.de (A.L.); ro.bissinger@gmx.de (R.B.); kashif_cbc@yahoo.com (K.J.)

**Keywords:** cell membrane scrambling, phosphatidylserine, calcium, cell volume, eryptosis

## Abstract

The selective cyclooxygenase-2 (COX-2) inhibitor celecoxib triggers apoptosis of tumor cells and is thus effective against malignancy. The substance is at least partially effective through mitochondrial depolarization. Even though lacking mitochondria, erythrocytes may enter apoptosis-like suicidal death or eryptosis, which is characterized by cell shrinkage and by phosphatidylserine translocation to the erythrocyte surface. Eryptosis may be triggered by increase of cytosolic Ca^2+^-activity ([Ca^2+^]_i_). The present study explored whether celecoxib stimulates eryptosis. Forward scatter was determined to estimate cell volume, annexin V binding to identify phosphatidylserine-exposing erythrocytes, hemoglobin release to depict hemolysis, and Fluo3-fluorescence to quantify [Ca^2+^]_i_. A 48 h exposure of human erythrocytes to celecoxib was followed by significant increase of [Ca^2+^]_i_ (15 µM), significant decrease of forward scatter (15 µM) and significant increase of annexin-V-binding (10 µM). Celecoxib (15 µM) induced annexin-V-binding was blunted but not abrogated by removal of extracellular Ca^2+^. In conclusion, celecoxib stimulates suicidal erythrocyte death or eryptosis, an effect partially due to stimulation of Ca^2+^ entry.

## 1. Introduction

The anti-inflammatory selective cyclooxygenase-2 (COX-2) inhibitor celecoxib [1,2] triggers apoptosis [1,2,3,4] and is thus considered for the treatment of malignancy [1,4,5]. The proapoptotic activity of the drug is apparently not the result of COX-2 inhibition [1,3] but at least partially due to decreased expression of Bcl-2 family members [4] and decreased mitochondrial potential [1,4]. Celecoxib further counteracts the anti-apoptotic proteins Mcl-1 and survivin [1]. Moreover, the drug has been shown to increase cytosolic Ca^2+^ activity ([Ca^2+^]_i_) [6]. The use of the drug is limited by its cardiovascular toxicity [1].

Cells like erythrocytes lacking mitochondria and nuclei should be insensitive to suicidal death triggered by mitochondrial depolarization and cytochrome c release [7]. Erythrocytes may, however, enter apoptosis-like suicidal death or eryptosis, which is characterized by cell shrinkage and phosphatidylserine scrambling of the cell membrane [7]. Eryptosis may be triggered by increase of [Ca^2+^]_i_. Ca^2+^ entry may be elicited by activation of Ca^2+^-permeable cation channels [8,9]. Stimulators of those channels include oxidative stress [10]. Increased [Ca^2+^]_i_ is followed by activation of Ca^2+^-sensitive K^+^ channels [11] causing cell shrinkage due to K^+^ exit, hyperpolarization, Cl^−^ exit and thus cellular KCl and water loss [12]. Increased [Ca^2+^]_i_ further stimulates cell membrane scrambling with phosphatidylserine exposure at the erythrocyte surface [13]. The Ca^2+^ sensitivity of cell membrane scrambling is enhanced by ceramide [14]. Eryptosis is further stimulated by energy depletion [15], caspase activation [16,17,18,19,20], and deranged activity of distinct kinases, such as AMP activated kinase AMPK [9], cGMP-dependent protein kinase [21], Janus-activated kinase JAK3 [22], casein kinase [23,24], p38 kinase [25], as well as sorafenib [26] and sunifinib [27] sensitive kinases.

Eryptosis is stimulated by a myriad of xenobiotics [28,29,30,31,32,33,34,35,36,37,38,39,40,41,42,43,44,45,46,47,48,49,50,51] and observed in several clinical disorders [7], such as diabetes [20,52,53], renal insufficiency [54], hemolytic uremic syndrome [55], sepsis [56], sickle cell disease [57], malaria [58,59,60,61,62], Wilson’s disease [62], iron deficiency [63], phosphate depletion [64], and presumably metabolic syndrome [51].

The present study explored the effect of celecoxib on [Ca^2+^]_i_, cell volume and phosphatidylserine abundance at the erythrocyte surface. As a result, the experiments disclose a powerful stimulating effect of celecoxib on eryptosis.

## 2. Results and Discussion

**Figure 1:**
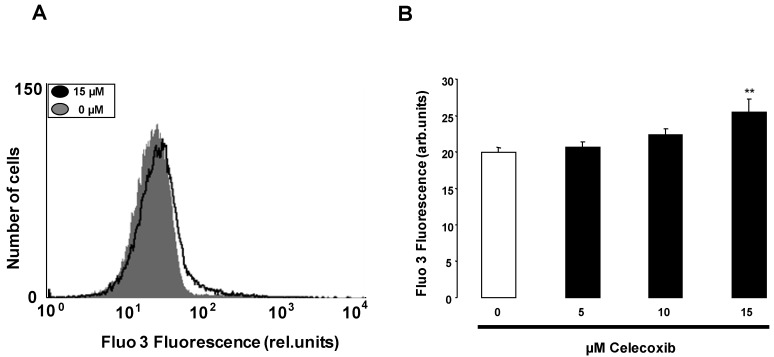
Effect of celecoxib on erythrocyte cytosolic Ca^2+^ concentration (**A**) Original histogram of Fluo3 fluorescence in erythrocytes following exposure for 48 h to Ringer solution (grey area) and with (black line) presence of 15 µM celecoxib; (**B**) Arithmetic means ± SEM (*n* = 10) of the Fluo3 fluorescence (arbitrary units) in erythrocytes exposed for 48 h to Ringer solution without (white bar) or with (black bars) celecoxib (5–15 µM). ** (*p* < 0.01) indicates significant difference from the absence of celecoxib (ANOVA).

The present study explored whether celecoxib triggers suicidal erythrocyte death or eryptosis, which is characterized by cell shrinkage and cell membrane scrambling, both events stimulated by increase of cytosolic Ca^2+^ activity ([Ca^2+^]_i_). In a first step, the effect of celecoxib on [Ca^2+^]_i_ was tested. To this end, human erythrocytes were loaded with Fluo3-AM and the Fluo3 fluorescence determined by flow cytometry. Prior to determination of Fluo3-fluorescence erythrocytes were incubated in Ringer solution without or with celecoxib (5–15 µM). As illustrated in Figure 1, a 48 h exposure of human erythrocytes to celecoxib resulted in an increase of Fluo3 fluorescence, an effect reaching statistical significance at 15 µM celecoxib concentration. Thus, celecoxib increased cytosolic Ca^2+^ concentration.

**Figure 2:**
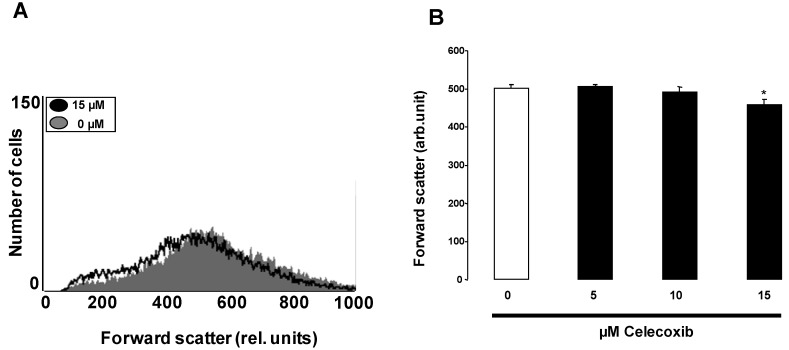
Effect of celecoxib on erythrocyte forward scatter. (**A**) Original histogram of forward scatter of erythrocytes following exposure for 48 h to Ringer solution without (grey area) and with (black line) presence of 15 µM celecoxib; (**B**) Arithmetic means ± SEM (*n* = 10) of the normalized erythrocyte forward scatter (FSC) following incubation for 48 h to Ringer solution without (white bar) or with (black bars) celecoxib (5–15 µM). * (*p* < 0.05) indicates significant difference from the absence of celecoxib (ANOVA).

An increase of [Ca^2+^]_i_ has been shown to activate Ca^2+^-sensitive K^+^ channels resulting in cell shrinkage due to KCl exit paralleled by osmotically obliged water. Cell volume was thus estimated from forward scatter determined in flow cytometry. As illustrated in Figure 2, a 48 h exposure to celecoxib led to a decrease of forward scatter, an effect reaching statistical significance at 15 µM celecoxib. Accordingly, celecoxib treatment was followed by erythrocyte shrinkage.

**Figure 3:**
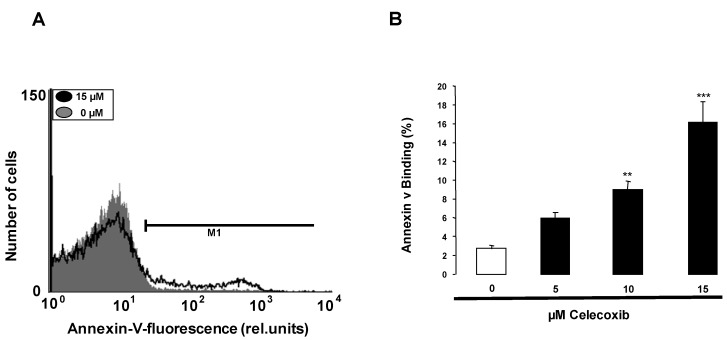
Effect of celecoxib on phosphatidylserine exposure and hemolysis*.* (**A**) Original histogram of annexin-V-binding of erythrocytes following exposure for 48 h to Ringer solution without (grey area) and with (black line) presence of 15 µM celecoxib; (**B**) Arithmetic means ± SEM of erythrocyte annexin-V-binding (*n* = 10) following incubation for 48 h to Ringer solution without (white bar) or with (black bars) presence of celecoxib (5–15 µM). ** (*p* < 0.01), *** (*p* < 0.001) indicate significant difference from the absence of celecoxib (ANOVA).

Increased [Ca^2+^]_i_ has further been shown to stimulate cell membrane phospholipid scrambling with phosphatidylserine exposure at the erythrocyte surface. To identify phosphatidylserine exposing erythrocytes annexin-V-binding was determined in flow cytometry. As shown in Figure 3, a 48 h exposure to celecoxib increased the percentage of annexin-V-binding erythrocytes, an effect reaching statistical significance at 10 µM celecoxib. Accordingly, celecoxib triggered erythrocyte cell membrane scrambling with phosphatidylserine exposure at the cell surface.

To explore whether celecoxib exposure triggers hemolysis, the percentage of hemolysed erythrocytes was estimated from hemoglobin concentration in the supernatant. As a result, the percentage of hemolysed erythrocytes approached 0.7% ± 0.2%, 2.5% ± 1.5%, 4.4% ± 1.8% and 9.6% ± 3.2% following exposure of erythrocytes for 48 h to 0, 5, 10, and 15 µM celecoxib (*n* = 4).

**Figure 4:**
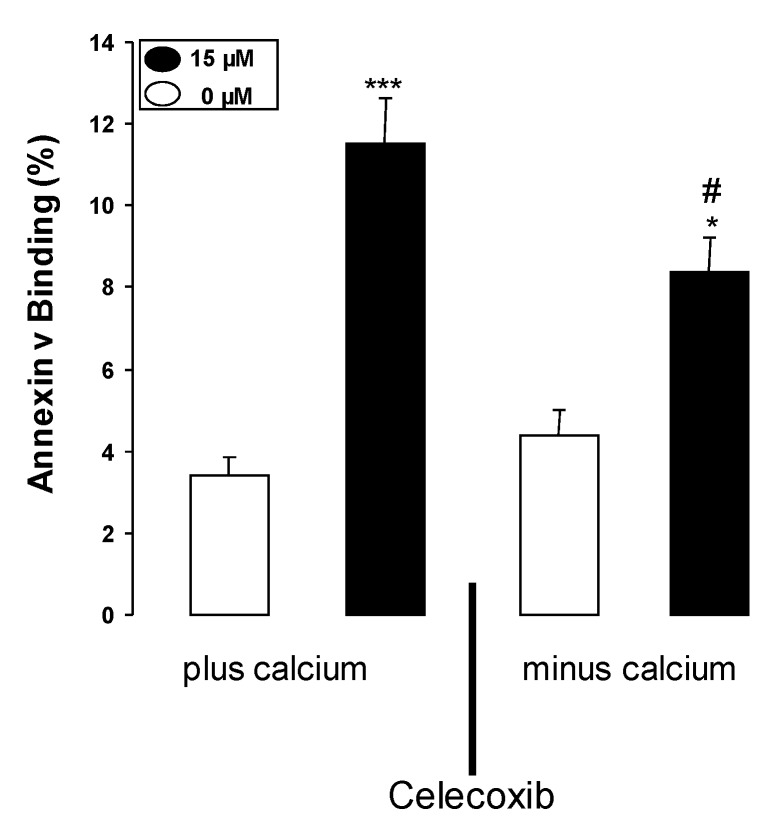
Effect of Ca^2+^ withdrawal on celecoxib-induced annexin-V-binding. Arithmetic means ± SEM (*n* = 6) of the percentage of annexin-V-binding erythrocytes after a 48 h treatment with Ringer solution without (white bar) or with (black bars) 15 µM celecoxib in the presence (left bars, Plus Calcium) and absence (right bars, Minus Calcium) of calcium. * (*p* < 0.05), *** (*p* < 0.001), indicate significant difference from the respective values in absence of celecoxib, # (*p* < 0.05) indicates significant difference from the respective value in the presence of Ca^2+^ (ANOVA).

In order to test, whether the celecoxib induced increase of [Ca^2+^]_i_ indeed contributed to or even accounted for the stimulation of celecoxib induced cell membrane scrambling, erythrocytes were exposed to 15 µM celecoxib for 48 h in the presence and in the nominal absence of extracellular Ca^2+^. As illustrated in Figure 4, the effect of celecoxib on annexin-V-binding was significatly blunted in the nominal absence of Ca^2+^. However, even in the nominal absence of extracellular Ca^2+^, celecoxib still significantly increased the percentage of annexin V binding erythrocytes. Thus, the effect of celecoxib was mainly but not exclusively due to Ca^2+^ entry.

The present study discloses a novel effect of celecoxib, *i.e.*, the stimulation of eryptosis, the suicidal death of erythrocytes. Treatment of human erythrocytes with celecoxib is followed by erythrocyte shrinkage and erythrocyte cell membrane scrambling, the hallmarks of eryptosis. The celecoxib concentrations required (10–15 µM) are similar to those (14.4–29.3) encountered *in vivo* [65].

The effect of celecoxib was paralleled by an increase of cytosolic Ca^2+^ activity, an effect paralleling a similar effect in nucelated cells [6]. The effect on annexin V binding was significantly blunted in the absence of extracellular Ca^2+^. Thus, the effect of celecoxib on cell membrane scrambling is at least in part due to stimulation of Ca^2+^ entry. Celecoxib presumably activates the Ca^2+^ permeable non-selective cation channels in erythrocytes. The molecular identity of those channels is incompletely defined but the channels involve the transient receptor potential channel TRPC6 [8]. Celecoxib presumably activates those channels possibly by inducing oxidative stress, which could be triggered by celecoxib [66,67] and is known to activate unspecific Ca^2+^ permeable cation channels in erythrocytes [10].

Ca^2+^ entry through the unspecific Ca^2+^ permeable cation channels further contributes to or even accounts for the celecoxib induced erythrocyte shrinkage. Erythrocytes express Ca^2+^ sensitive K^+^ channels [11,68], which are activated by increase of cytosolic Ca^2+^ activity. Activation of those channels results in cell shrinkage due to K^+^ exit, cell membrane hyperpolarisation, Cl^−^ exit and thus cellular loss of KCl with osmotically obliged water [12].

The stimulating effect of COX-2 inhibitor celecoxib is in seeming contrast to the effect of unselective COX inhibitors observed earlier. Osmotic cell shrinkage has been shown to trigger the release of PGE_2_, which in turn activates the unspecific cation channels and thus triggers Ca^2+^ entry and suicidal erythrocyte death [69]. In the presence of unspecific COX inhibitors Ca^2+^ entry and suicidal erythrocyte death following hyperosmotic shock were significantly blunted. Presumably, the Ca^2+^ entry and suicidal erythrocyte death observed following exposure of erythrocytes to celecoxib is not due to inhibition of PGE_2_ formation but due to an unrelated side effect of the drug.

Phosphatidylserine exposing erythrocytes adhere to endothelial CXCL16/SR-PSO of the vascular wall [70]. The adherence of the phosphatidylserine exposing erythrocytes to the vascular wall presumably interferes with blood flow [70,71,72,73,74,75]. Thus, eryptosis may be expected to impair microcirculation. Moreover, phosphatidylserine exposure of erythrocytes fosters blood clotting and may thus cause thrombosis [71,76,77], a side effect observed following celecoxib treatment [78].

Phosphatidylserine exposing erythrocytes are further rapidly cleared from circulating blood [7]. If the accelerated loss of erythrocytes cannot be outweighed by compensating increase of erythrocyte formation, the stimulation of eryptosis may lead to anemia [7], again a known side effect of celecoxib [79].

## 3. Methods

### 3.1. Erythrocytes, Solutions and Chemicals

Leukocyte-depleted erythrocytes were kindly provided by the blood bank of the University of Tübingen. The study is approved by the ethics committee of the University of Tübingen (184/2003 V). Erythrocytes were incubated *in vitro* at a hematocrit of 0.4% in Ringer solution containing (in mM) 125 NaCl, 5 KCl, 1 MgSO_4_, 32 *N*-2-hydroxyethylpiperazine-*N*-2-ethanesulfonic acid (HEPES), 5 glucose, 1 CaCl_2_; pH 7.4 at 37 °C for 48 h. Where indicated, erythrocytes were exposed to celecoxib (Sigma, Freiburg, Germany) at the indicated concentrations. In Ca^2+^-free Ringer solution, 1 mM CaCl_2_ was substituted by 1 mM glycol-bis(2-aminoethylether)-*N*,*N*,*N*',*N*'-tetraacetic acid (EGTA).

### 3.2. FACS Analysis of Annexin-V-Binding and Forward Scatter

After incubation under the respective experimental condition, 50 µL cell suspension was washed in Ringer solution containing 5 mM CaCl_2_ and then stained with Annexin-V-FITC (1:200 dilution; ImmunoTools, Friesoythe, Germany) in this solution at 37 °C for 20 min under protection from light. In the following, the forward scatter (FSC) of the cells was determined, and annexin-V fluorescence intensity was measured with an excitation wavelength of 488 nm and an emission wavelength of 530 nm on a FACS Calibur (BD, Heidelberg, Germany).

### 3.3. Measurement of Intracellular Ca^2+^

After incubation erythrocytes were washed in Ringer solution and then loaded with Fluo-3/AM (Biotium, Hayward, CA, USA) in Ringer solution containing 5 mM CaCl_2_ and 2 µM Fluo-3/AM. The cells were incubated at 37 °C for 30 min and washed twice in Ringer solution containing 5 mM CaCl_2_. The Fluo-3/AM-loaded erythrocytes were resuspended in 200 µL Ringer. Then, Ca^2+^-dependent fluorescence intensity was measured with an excitation wavelength of 488 nm and an emission wavelength of 530 nm on a FACS Calibur (BD, Heidelberg, Germany).

### 3.4. Measurement of Hemolysis

For the determination of hemolysis the samples were centrifuged (3 min at 400*g*, room temperature) after incubation, and the supernatants were harvested. As a measure of hemolysis, the hemoglobin (Hb) concentration of the supernatant was determined photometrically at 405 nm. The absorption of the supernatant of erythrocytes lysed in distilled water was defined as 100% hemolysis.

### 3.5. Statistics

Data are expressed as arithmetic means ± SEM. As indicated in the figure legends, statistical analysis was made using ANOVA with Tukey’s test as post-test and *t* test as appropriate. n denotes the number of different erythrocyte specimens studied. Since different erythrocyte specimens used in distinct experiments are differently susceptible to triggers of eryptosis, the same erythrocyte specimens have been used for control and experimental conditions.

## 4. Conclusions

Celecoxib triggers cell shrinkage and cell membrane scrambling of human erythrocytes, an effect at least partially due to stimulation of Ca^2+^ entry. Celecoxib is thus able to trigger suicidal death of erythrocytes, *i.e.*, cells devoid of mitochondria and nuclei.
